# Meat Safety in Northern Tanzania: Inspectors' and Slaughter Workers' Risk Perceptions and Management

**DOI:** 10.3389/fvets.2020.00309

**Published:** 2020-06-18

**Authors:** Linda Waldman, Tabitha A. Hrynick, Jackie Benschop, Sarah Cleaveland, John A. Crump, Margaret A. Davis, Boniface Mariki, Blandina T. Mmbaga, Niwael Mtui-Malamsha, Gerard Prinsen, Joanne Sharp, Emmanuel S. Swai, Kate M. Thomas, Ruth N. Zadoks

**Affiliations:** ^1^Institute of Development Studies, University of Sussex, Brighton, United Kingdom; ^2^^*m*^EpiLab, School of Veterinary Science, Massey University, Palmerston North, New Zealand; ^3^Institute of Biodiversity, Animal Health and Comparative Medicine, College of Medical, Veterinary and Life Sciences, University of Glasgow, Glasgow, United Kingdom; ^4^Nelson Mandela African Institution of Science and Technology, Arusha, Tanzania; ^5^Centre for International Health, Dunedin School of Medicine, University of Otago, Dunedin, New Zealand; ^6^Kilimanjaro Christian Medical University College, Tumaini University, Moshi, Tanzania; ^7^Kilimanjaro Christian Medical Centre, Moshi, Tanzania; ^8^Paul G. Allen School for Global Animal Health, College of Veterinary Medicine, Washington State University, Pullman, WA, United States; ^9^Tanzania Chamber of Commerce, Moshi, Tanzania; ^10^Kilimanjaro Clinical Research Institute, Good Samaritan Foundation, Moshi, Tanzania; ^11^Ministry of Livestock and Fisheries, Dodoma, Tanzania; ^12^School of People, Environment and Planning, Massey University, Palmerston North, New Zealand; ^13^School of Geographical and Earth Sciences, University of Glasgow, Glasgow, United Kingdom

**Keywords:** meat safety, risk perception, Tanzania, slaughter, foodborne disease, *Salmonella*, *Campylobacter*

## Abstract

Through a social scientific lens, this paper considers the risk perceptions and “risk-based decision-making” of two key groups in a northern Tanzanian context: (1) frontline government meat inspectors and health officers charged with ensuring that red meat sold commercially is safe for people to consume, and (2) the workers who slaughter and process cattle and red meat prior to its sale in rural butcheries. In contrast to techno-scientific understandings of disease risk and “rational” approaches to its management, this paper foregrounds the role of social, economic and institutional context in shaping the perceptions and practices around meat safety of these actors whose daily, close proximity to meat means they play a significant role in mitigating potential meat-borne disease. We show how limited resources, and a combination of scientific and local knowledge and norms result in “situated expertise” and particular forms of risk perception and practice which both enhance and compromise meat safety in different ways. Actors' shared concerns with what is visible, ensures that visibly unsafe or abnormal meat is excluded from sale, and that infrastructure and meat is kept “clean” and free of certain visible contaminants such as soil or, on occasion, feces. While such contaminants serve as a good proxy for pathogen presence, meat inspectors and especially slaughter workers were much less aware of or concerned with invisible pathogens that may compromise meat safety. The role of process and meat handling did not figure very strongly in their concerns. Microorganisms such as *Salmonella* and *Campylobacter*, which can easily be transferred onto meat and persist in slaughter and meat sale environments, went unacknowledged. Although health officers expressed more concern with hygiene and meat handling, their influence over slaughter process and butchery operations was unclear. Ultimately, recognizing the perceptions and practices of frontline actors who engage with meat, and the ways in which social, material and institutional realities shape these, is important for understanding how decisions about risk and meat safety are made in the complexity and context of everyday life, and thus for finding effective ways to support them to further enhance their work.

## Introduction

Tanzania hosts Africa's third largest national population of livestock, upon which millions at least partially depend for their livelihoods. Despite policies to modernize the livestock sector through improved and intensified farming mechanisms, it is estimated that 88% of cattle in the country continue to be held in small-scale peri-urban farms and/or large-scale traditional pastoral systems. Such cattle provide the majority of domestically consumed cattle-derived food products, as population growth and rising per capita income increase demand (1–3). Despite the presence of urban abattoirs and some rural slaughterhouses, many cattle destined for slaughter and for the commercial sale of meat are killed at small, rural, concrete slaughter slabs, usually owned by local butchers, where they are slaughtered by a few workers with simple tools (such as knives, cleavers and ropes)^1^. Before meat is transported to butcheries, it must be inspected by a certified government meat inspector (MI) and stamped to indicate fitness for human consumption, or otherwise condemned. MIs (many of whom also provide livestock extension services), alongside health officers (HOs), are also charged with ensuring infrastructural and hygiene standards at slabs and in butcheries are met.

Emerging evidence suggests that the extent and burden of food-borne disease (FBD), including meat-borne disease, in low- and middle-income countries (LMICs) is substantial. A landmark WHO study concluded that Africa suffers the highest per capita burden of FBD globally (5), and the World Bank recently estimated FBD costs LMICs at least 110 bn USD annually (6). Many FBDs, such as salmonellosis, can be transmitted from animals to humans through the handling or consumption of meat and other animal products (although contamination of animal products by human or environmental pathogens is also possible).

Explanations for why LMICs have high burdens of FBD frequently highlight the predominance of small, informal actors, poor infrastructure, and weak regulation and capacity for enforcement in LMICs (7). Lack of awareness of presence and transmission risks of disease-causing organisms among food handlers and consumers is also oft cited, but numerous studies have demonstrated that provision of the “right” information does not necessarily lead to behavior change or adoption of more “rational” risk-based decision-making in relation to food safety, whether in low, middle or high income countries (8). Rather, perspectives from the social sciences highlight how people's risk perceptions and behaviors in all contexts, are highly situated and shaped by a range of psychological, social and economic factors (9).

This paper offers a rare social science contribution to the study of risk-based decision making in relation to meat safety in a low-income country context. Little information on risk perception in LMICs is available, which creates an opportunity to take greatest advantage of qualitative analysis. Through observation and semi-structured interviews, which “communicate experiences and opinions in an articulate, expressive and reflective manner” (10) and are therefore excellent for eliciting rich and candid responses from interviewees, this paper explores the perceptions of meat safety and its management among two key sets of actors in Tanzania. These are: (1) state employees with direct roles in ensuring meat safety, namely MIs and HOs, (together henceforth referred to as inspectors) and (2) people involved in the slaughter process at small slaughter slabs and slaughterhouses in rural areas, many of whom also own and/or work in small local butcheries (hereafter termed slaughter workers). By approaching “risk” as a variable, socially constructed notion, rather than an objective scientific measure of probability that legitimates particular technical control measures, this paper presents a contextually embedded analysis of how meat safety actually “happens” in the complexity of the everyday lives of these “risk managers” (11). In doing so, we recognize the broader social economic systems and conditions in which inspectors and slaughter workers operate, and how for instance, available resources influence their perceptions, inclinations and capacities to act (12, 13). More specifically, we illustrate how local conceptualizations of risk emphasize animal health, the appearance of meat, and certain infrastructural and hygienic aspects of slaughter and butchery environments while downplaying others, such as how meat is handled. Such perspectives help to elucidate the strengths of existing practice, as well as areas where contextually embedded risk managers, like the inspectors and slaughter workers considered here, can be supported to further enhance meat safety in realistic and appropriate ways. These insights are vital for meat safety policy in LMICs, but can also potentially inform high-income countries where those who work in close proximity to meat may also hold different forms of knowledge and are subject to variabilities of context.

### Background: Understanding and Managing Food Safety

The intensification of production, the complex elongation of value chains and the rise in demand for animal products has led to increasing concern over the possibilities of food safety breaches with potentially devastating and far-reaching effects [cf. (14)]. In high-income countries, governments and private industry actors have reoriented meat safety policies and practice toward risk-based preventative approaches seen to be more effective, particularly against pathogens and hazards undetectable through traditional sensory inspection regimes (such as *Salmonella* and *Campylobacter*) (15–17). However, highly formalized preventative risk-based approaches, such as Hazards Analysis and Critical Control Points (HACCP) and Good Management Practices (GMPs), have not proven easily adoptable for smaller scale operations, or for producers in low-resource contexts (18–20). Yet, food safety policy and regulation in LMICs is often modeled on international and regional standards, export markets and “best practices” associated with particular food value chains. In addition, governments are urged to undertake their own “risk analysis.” This involves expert “risk assessors” identifying hazards, quantifying and comparing their prevalence, probabilities and impacts, and possibly testing control measures. In combination with scientific understandings of how particular pathogens or contaminants enter and move through value chains, this information is seen as crucial to ensuring food safety, as is its effective communication to “risk managers” who, armed with this “objective data,” can make “rational decisions” about biosecurity and control (11, 21, 22). “Risk managers”—in this paper considered to be those government employees undertaking day-to-day assurance of food safety, as well as private sector actors engaged in small-scale slaughter and raw meat sale—are expected to take decisions in accordance with this technical risk knowledge as it is embedded in policy (23). These expectations however, rely on a set of assumptions, including that such information reaches relevant actors, that they understand, accept and prioritize the information in expected ways, and that they have the capacity to act on it.

Social science approaches recognize “risks” as nebulous, socially variable notions of what is hazardous, of cause and effect, and of whether and how control should occur or caution be taken (24). Research in the cognitive psychology tradition has drawn a distinction between “expert” and “lay” perceptions of risk, emphasizing in relation to the latter, “affective” human responses to different characteristics of particular risks such as voluntariness, controllability, or dread. For instance, Jensen et al. illustrated in 2005 that experts perceived lay concern in Denmark over bovine spongiform encephalitis (BSE) as “irrational” because it diverted attention and resources away from *Salmonella* prevention which, while less immediately frightening to the public, was far more common and had significant, if diffuse, societal impacts (25). Anthropological approaches to risk have highlighted the socially constructed nature of risk perceptions, situating “risk” within social, cultural, economic, and political systems (9, 26–28). In other words, people's risk perceptions, responses to risks, and even “risks” themselves are shaped by interacting and dynamic contextual factors and constraints. Furthermore, risk perceptions can vary considerably from person to person in the same context. Considering risk from a primarily technical perspective and failing to take socio-cultural contexts, structural realities, and resources and power into account, has the effect of casting blame upon individuals and/or cultures for their own vulnerability to, or role in generating, risk (29).

While structural and contextual factors shape risk perceptions and responses in key ways, it is also important to recognize individual agency (30). With their formal training^2^ on public health risk and associated regulations and specific responsibility for meat safety, inspectors in northern Tanzania occupy uniquely powerful positions through which they can and do mitigate meat safety risks, sometimes in unexpected ways. Furthermore, as “street-level bureaucrats,” these government staff have considerable discretion, often bringing their own values, priorities and understandings to bear when implementing policy (33, 34). This has been framed both as problematic divergence from high-level policy goals, but also, as creative and necessary to operate effectively in complex, messy realities—especially in the face of limited resources (35, 36). Slaughter workers also have substantial influence over meat safety given their daily activities of slaughter and dressing, and based on their own knowledge, concerns and priorities, deploy their own forms of risk-based decision-making. While they learn some elements of technical risk knowledge and control through their contact with inspectors, who also explain what regulations they must follow, slaughter workers' understandings and management of risk are also shaped by on-the-job experience and a wide range of other influences. As Sjölander-Lindqvist and Cinque (12) argue, personal experiences, feelings and beliefs inform decision-making even in situations where decision-makers are expected to rationally assess the effects of their choices and strategies. In addition to being an emotionally complex process which draws on individuals' experience and intuitive knowledge, it is also often a shared, collaborative activity in which individuals make assumptions about the commitments, priorities and assumptions of others. It is a fluid social phenomenon, shaped by “contextualized processes of interaction between individuals, authorities, and social structures” (37).

Despite the daily and intimate interfacing between inspectors, slaughter workers and meat and thus the importance of understanding the ways in which these actors perceive, construct, prioritize, manage and make decisions about risk in relation to meat safety, research has tended to focus on the risk perceptions of meat consumers (38–40). This paper expands our understanding of inspectors' and slaughter workers' perceptions, priorities and practices in relation to meat safety in northern Tanzania, asking what lies behind them, and what this might mean for meat safety and technological paradigms of risk management.

## Methods

This paper is based on two sets of relatively open-ended, semi-structured interviews. This type of open-ended interview, often used in qualitative research, encourages respondents to share their experiences and viewpoints (10) in ways that reveal information not usually sought in structured surveys or questionnaires, thereby creating opportunities for unanticipated insights to emerge both in the interviews and through the analysis (41). The interviews were conducted by a Tanzanian interviewer (BM) in northern Tanzania as part of a multi-disciplinary project to understand hazards associated with zoonotic enteric pathogens in emerging livestock meat pathways (HAZEL). One set (*n* = 19), taking place between February 2017 and February 2018 was conducted with MIs (*n* = 10), and HOs (*n* = 9), the latter of whom are also charged with ensuring food establishments, including butcheries, comply with food safety standards through inspection and enforcement^3^. Half were conducted with respondents from five urban wards in Moshi Municipality, and half were conducted in five rural wards of Moshi District, both in the Kilimanjaro Region of Tanzania's Northern Zone. Moshi, in north eastern Tanzania, was chosen because it offered the possibility to study both traditional and emerging livestock meat pathways in an agro-ecological setting (with scope to explore urban, peri-urban, mixed crop and livestock and pastoral-wildlife interfaces) as well as providing opportunities to work with policy actors to identify areas for improvement in food safety policy and practice in Tanzania. Respondents were asked about their work and duties, challenges, perceptions of policy, experiences with animal disease and meat safety, and related expectations and recommendations for the future. Where meaningful differences between the responses of urban and rural inspectors have been observed, this is noted in the paper.

The other set of interviews was conducted with people working at slaughter slabs (*n* = 13) and slaughterhouses (*n* = 2) in the same five rural wards in northern Tanzania in which rural inspectors were interviewed, between August 2017 and September of 2018. These respondents had between one and 30 years of experience as slaughter workers, with only three having <7 years' experience. Cattle were the primary animals slaughtered at these sites. Most slaughter workers explained that the most affordable, healthy animals were purchased by butchery owners or their representatives from traders at cattle markets in the region (they were less sure where animals originated prior to auction), and occasionally from local farmers. The decision to focus on rural wards was based on the observation that rural sites, and the inspectors who serve them, face potentially greater challenges (such as long distances between sites) than their urban counterparts (36). All slaughter workers interviewed were directly involved in slaughter activities, and ranged from cleaners, skinners, and slaughterers, to slab owners who frequently also owned and operated butcheries (often adjacent to slabs). Some slabs were used by more than one butcher, and were sometimes rented to others on agreed days and times. Two respondents from rural slaughterhouses were interviewed, namely a manager who oversaw operations at a government-owned facility, and an individual who carried out the majority of slaughter work and cleaning in a smaller, privately-owned slaughterhouse. Butchers operating within reasonable distance from slaughterhouses were expected to have their animals slaughtered in these facilities, while those beyond reasonable distance used their own slabs. These interviews included questions on slaughter workers' routines and practices, aspects of the slaughter environment, its management and change over time, understandings of meat safety, and relationships with inspectors.

Interview participants were selected to cover a range of workers fulfilling a diversity of roles associated with meat and slaughter. The interviews attempted to capture contextually rich depictions of respondents' routines, experiences, perceptions, priorities and practices in the complex context of everyday life (24) as a central premise of this research is that these aspects are constitutive of “decision-making” and cannot be understood independently of this. As researchers, we draw on anthropological understandings that highlight the complex, fluid, interactional and situated dimensions associated with decision-making rather than reifying rational and calculated individualized choices (12, 37). All interviews were conducted, recorded and transcribed in Kiswahili before being translated into English by the interviewer (BM). Field notes were also taken by researchers (GP, LW, TH) during visits to the study area between March 2015 and March 2018. Data analysis of the interviews was conducted through a primarily inductive approach by the authors (TH, LW). Interviews were thoroughly read to gain a sense of overarching themes related to our research questions:

1. What are the perceptions, priorities, and practices of inspectors and slaughter workers in relation to risk and meat safety?
2. What might explain these perceptions, priorities and practices?

An initial coding structure was created based on these broad themes and then interviews were coded in an iterative, cyclical process using NVivo 12 (QSR International, Australia). The coding structure developed and evolved as familiarity with the data deepened and new patterns and connections were noticed (41). This process was documented in internal analytical memos. Quantitative observational data (noting infrastructural provision and hygiene practices) collected during visits to nine of the slabs and both slaughterhouses, are triangulated with qualitative observations in this paper.

Research was approved by the Tanzanian National Institute of Medical Research (Ref. NIMR/HQ/R.8a/Vol. IX/2028 and extension Ref. NIMR/HQIR.8cNol. 11/1069); the Kilimanjaro Christian Medical Centre (KCMC) Ethics Committee (Research Ethical Certificate No. 832); the Ethics Committee of the College of Medical, Veterinary and Life Sciences at the University of Glasgow, Glasgow, UK (Refs. 200140183 and 200140152) and the Human Research Ethics Committee at the University of Otago, Dunedin, New Zealand (Ref. H15/069). In accordance with the Ethical Approval process and documentation and the standard “Framework for Research Ethics” produced by the UK Economic and Social Research Council, interviewees gave recorded, verbal consent to participate. Researchers ensured that respondents knew that their involvement was entirely voluntary, and that they could terminate their involvement at any point.

## Results

This section presents respondents' understandings, priorities and practices, and thus their decision-making, —in relation to risk and meat safety under three major themes: (1) unsafe meat and disease; (2) infrastructure and equipment; and (3) hygiene and cleanliness.

### Unsafe Meat and Disease

One indicator of respondents' risk perceptions and priorities in relation to meat safety was the set of pathogens or diseases they referred to during their interviews (see Table 1). Anthrax, endemic in East Africa, and causing visible abnormalities to carcasses and meat as well as recognizable symptoms in humans, was foremost in respondents' minds. It was mentioned 80 times by 16 of 19 inspectors—more than four times as much as African swine fever, the next most frequently mentioned disease (which is not zoonotic and does not pose a direct threat to human health). Although slaughter workers primarily processed cattle, inspectors' activities were not limited to cattle as reflected by mention of diseases affecting other animal species (rabies, African swine fever, Newcastle disease).

**Table 1:** Specific diseases and conditions mentioned by inspectors and slaughter workers.

	**Inspectors (*****n*** **=** **19)**		**Slaughter workers (*****n*** **=** **15)**	
**Diseases/pathogens mentioned**	**Times mentioned**	**No. respondents mentioning this**	**Times mentioned**	**No. respondents mentioning this**
Anthrax	80	16	14	8
African swine fever	18	5	0	0
Rabies	16	9	0	0
Tuberculosis (or “TB”)	16	8	0	0
Brucellosis	6	3	0	0
Liver flukes/Fasciola	6	5 (only 1 used “*Fasciola*”)	1 (“fluke”)	1
*Cysticercus bovis*	8	4	1 (colloquial term: “*fini*”)	1
Foot and mouth disease	6	3	0	0
Liver cirrhosis	6	2	1	1
Newcastle disease	5	3		
Ebola	2	1	0	0
Trypanosomiasis	3	2	1 (“sleeping sickness”)	1

Inspectors recounted experiences with anthrax when asked about their successful prevention of animal-to-human disease transmission. These experiences included attending to a specific case after hearing about a suspicious livestock death from community members, and arriving at a slaughter slab for inspection and finding that workers had slaughtered an infected animal:^4^

*We discovered this when the animal was slaughtered and I observed the blood. The owner claimed the animal was crushed by others in a truck from auction. The blood didn't clot. I gave an order to stop the skinning and took a sample for diagnosis. It was confirmed to be anthrax. I immediately condemned the animal and dug a deep hole to bury it with lime to prevent bacteria migrating to the surface*. (urban MI)

*The animal died suddenly and people decided to butcher it […]. They thought it died from a normal disease. I was informed and went to see it. You know anthrax has very obvious signs. The blood was still fresh, I quickly understood it was anthrax. I stopped all procedures. They were lucky we responded quickly. Some were taken to hospital and the carcass was condemned and buried*. (rural HO)

In addition to describing real-life encounters with the disease, anthrax was also frequently used to illustrate hypothetical situations, and respondents' corresponding responsibilities in relation to meat safety.

*My role is to inspect meat at slaughter sites and certify it is safe to eat. If not, I will condemn it. If it has anthrax for example, I won't allow the carcass out of the area. All people involved will be required to go for treatment at the nearest dispensary. The meat will be condemned and buried*. (rural MI)

Inspectors emphasized that anthrax was “*very obvious”* and easy to identify and they frequently drew on anthrax signs when explaining how they knew meat was unsafe for consumption.

Anthrax was also the most commonly referenced specific disease among slaughter workers, who mentioned it disproportionately to other animal diseases, with eight out of 15 referencing it 14 times (see Table 1). Four described signs of anthrax, while two noted they had learned to identify it from inspectors.

*We have been taught by the MI that, if the blood doesn't clot after slaughter, not to touch the animal and to wash our hands with kerosene. It has anthrax*. (rural slaughter worker)

When asked what they felt the future held for zoonotic disease and meat safety, most inspectors (*n* = 16) and slaughter workers (*n* = 12) reported that disease would continue to decline. This conclusion was “evidenced” by the perception that incidents and outbreaks of anthrax had slowed or ceased.

*I think major problems regarding zoonotic diseases are declining. For example, this year I have not heard of any serious animal disease outbreak like anthrax*. (rural MI)

In addition to anthrax, inspectors also expressed concerns about a range of different types and degrees of health risks, including bovine tuberculosis (bTB), brucellosis and *Cysticercus bovis* among others. While slaughter workers did not often name particular diseases, almost all (*n* = 13) were confident they could tell when live animals were ill. “*Lungs that bulge out,”* “*standing hair*,” unusual breathing or salivation, and weakness were cited as indications.

Many slaughter workers also claimed to be able to identify inner organs which looked “*unusual*,” such as with “*swells and accumulations of fluids”* or having “*threads or worms.”* Visibly diseased—even “*completely destroyed*”—livers, lungs and kidneys were considered unsafe for human consumption and it was recognized that these would be condemned by the inspectors.

*Given our experience, we can see when an organ appears differently. You know some animals drink contaminated water from ponds and they become sick…We can see things like worms in the intestine and other organs. When the MI comes, we tell him what we have seen*. (rural slaughter worker)

As illustrated in the above examples, MIs and slaughter workers emphasized visible signs of disease when considering meat safety, and well-known and easily visually identified diseases like anthrax. Invisible organisms such as *Salmonella* and *Campylobacter*, which are often present as commensals in the digestive systems of healthy cattle but do not cause abnormalities in meat or organs, and can be introduced onto meat through slaughter and handling, went unmentioned. As shown in the above quote and Table 2, when asked what *caused* meat to become unsafe, slaughter workers primarily identified animal disease caused by livestock consuming “*poisonous*” food or “*dirty*” water, particularly at cattle markets or in transit, or by livestock keepers' poor practices such as failure to vaccinate and treat animals.

*Animals come with infections from the source. There is no problem here. After all, they stay for only a few days before being slaughtered*. (rural slaughter worker)

Although not specifically asked about the causes of unsafe meat, many inspectors similarly associated livestock keepers and their practices (lack of vaccination; poor treatment of animals; low levels of awareness; poverty; and/or unwillingness to make investments) with animal disease, and thus, unsafe meat.

**Table 2:** Slaughter workers' responses to the question “what causes meat to become unsafe for people to eat”?

**What causes meat to become unsafe for people to eat?**	**Number of slaughter workers reporting this (*n* = 15)**
Animal drinking contaminated/standing water	8
Animal consuming grass, bad food, or grazing in the bush	7
Livestock keeper practices	7
Animal exposed to insect vectors	2
Contact with other animals	2
Don't know	1
Starvation	1
Climate change	1
Slaughtering, skinning and chopping	1

**Figure 1:**
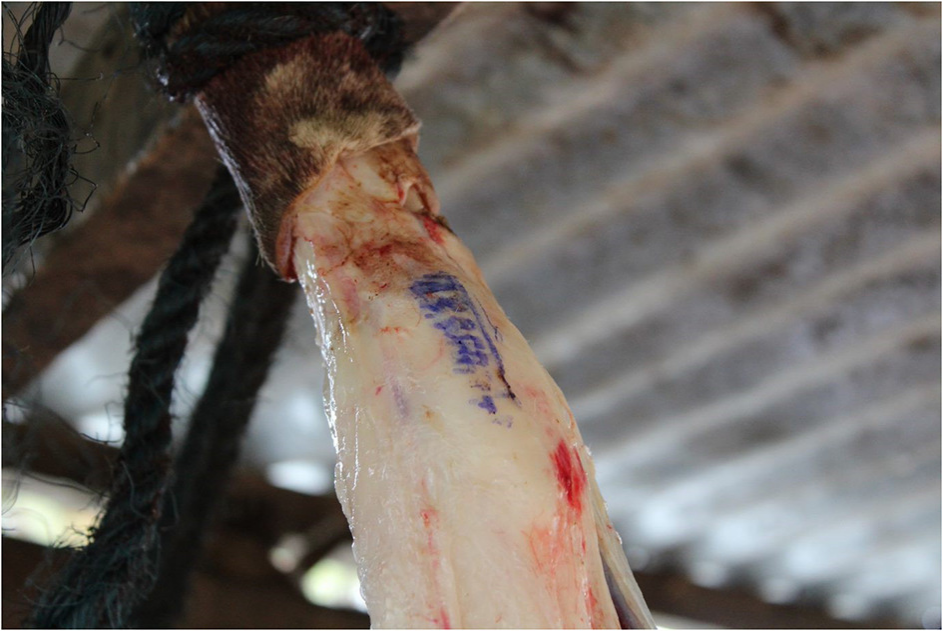
A government stamp indicating that meat from this carcass was inspected and thus deemed safe for human consumption. Photo: Mary Ryan.

When asked what they did to ensure meat from their establishments was safe for human consumption, slaughter workers' most frequent response was that they ensured meat was inspected and marked safe with a government stamp by an official MI (see Figure 1 and Table 3, discussed in more detail below). They regarded MIs as experts, trusted their judgements, and saw the stamp as an important visual signifier that their meat was safe.

*We have no reported case of affected customers after buying meat from our butchery. This has not happened because we rely on the MI's report after inspection. We don't sell uninspected meat*. (rural slaughter worker)

MIs also emphasized their meat inspection duties. Indeed, all devoted more time and detail to describing this aspect of their responsibilities at the outset of the interview (*vis a vis* their roles as providers of livestock extension services and, as discussed in the next section, as enforcers of infrastructural and equipment standards). Cross checking for the stamp in their own inspections (*n* = 6) or encouraging customers not to purchase unstamped meat (*n* = 2) was also mentioned by HOs.

**Table 3:** Number of inspectors indicating they enforced particular infrastructural or equipment provisions, and/or pointed to their adoption as evidence of positive change.

**Infrastructure/equipment**	**Urban MIs (*n* = 5)**	**Rural MIs (*n* = 5)**	**Urban HOs (*n* = 5)**	**Rural HOs (*n* = 4)**
Tiles on floors, walls, counters	5	5	4	4
Glass windows/doors	5	4	3	2
Uniforms	5	2	5	3
Water available (not necessarily running water)	3	3	3	4
Plastic chopping boards	5	1	4	0
Handwashin*g* facilitie*s*	0	2	2	3
Meat saws	1	2	4	0
Ceiling boards	1	0	1	1
Freezers	1	0	0	0

### Infrastructure and Equipment

A number of Tanzanian national laws outline the responsibilities of government ministries to draw up regulations “for any matter in relation to slaughter and slaughter facilities which appears […] necessary for the proper maintenance of quality standards in respect of meat intended for human consumption”^5^. In addition to requiring ante- and post-mortem inspections, there are infrastructural, procedural, and personnel standards to be followed in premises where slaughter or meat sale occur. While not always in possession of published regulations, inspectors seemed clear about their responsibilities which, they assured us, were clearly spelled out in by-laws and “directives”^6^.

As interviews progressed, MIs emphasized their responsibilities and efforts beyond meat inspection to ensure that certain elements of physical infrastructure were present and that particular pieces of equipment were used by workers. HOs also mentioned these provisions. In relation to butcheries, such infrastructural standards included easy to clean tiled walls and floors, glass doors and windows (to prevent flies and dust), and although less frequently mentioned, facilities for hand washing. Urban inspectors were particularly concerned with staff uniforms and plastic chopping boards (although the appropriateness of the latter was questioned by some).

When asked about whether there had been any change in slaughter or meat sale practices in the last 5–10 years, inspectors frequently pointed to improved infrastructure and adoption of equipment as evidence of positive change. Table 3 shows the number of inspectors who claimed to enforce or encourage infrastructural elements or equipment in butcheries, or highlighted their adoption as evidence of positive change over the past 5–10 years.

**Figure 2:**
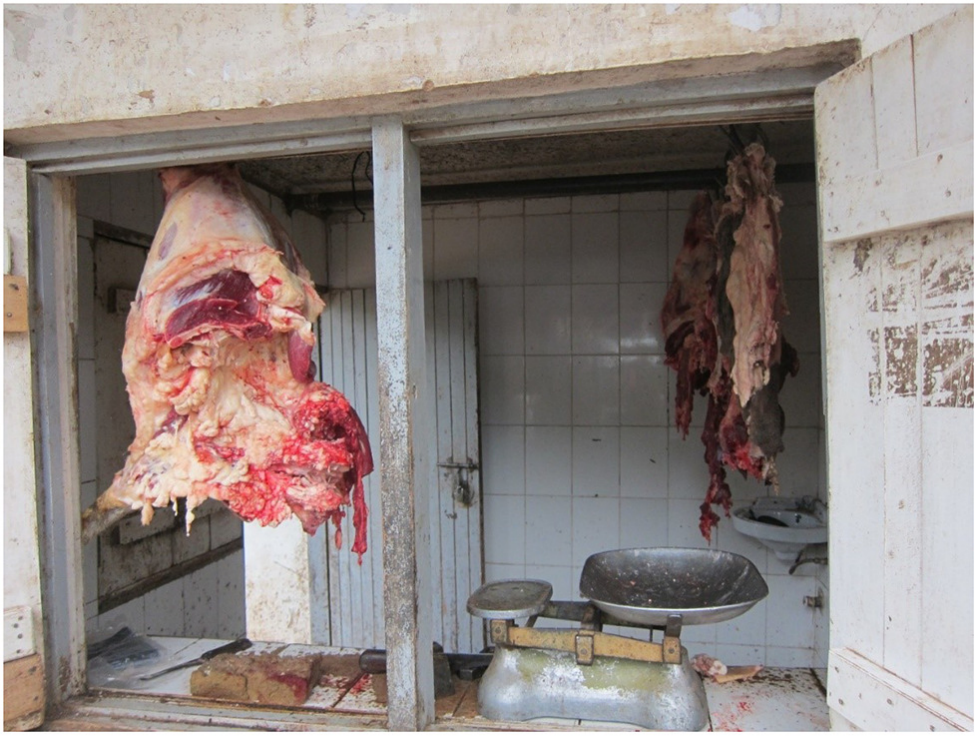
This rural butchery, situated in a permanent structure, featured a tiled counter, walls and floors and even a sink, but no running water. We also observed few rural butcheries with glass windows. Photo: Tabitha Hrynick.

Butcheries which made these legislated upgrades—built permanent structures, tiled walls, and installed glass doors and windows or other screens—were described as “*modern,”* “*clean,”* and “*attractive”* (see Figure 2). They were contrasted with “*dirty,” “very simple”* structures of the past—temporary wooden shacks or meat simply hung from tree branches for sale—and by implication, were seen as more facilitative of meat safety.

*You can see newly-constructed, rehabilitated slabs and butcheries. Good number of butcheries now have tiles and are very modern. The old dirty butcheries are no longer there […]* (rural MI)

Getting butcheries to make these upgrades was described by inspectors as a slow and ongoing process which they perceived to be largely the result of their active, consistent enforcement and efforts to persuade butchers, and their ability to eventually “*use force*” to close the establishment if necessary.

*When a new directive comes, the response is slow. Not much change is done voluntarily. […] When we asked them to fix tiles and glass windows and doors they didn't understand. They complained it was too costly. But after some months of strong follow-up, they responded as you can see*. (rural HO)

As suggested in the above quote, inspectors recognized that butchers resisted or could not easily afford to make expected changes, and thus gave them time to adapt.

As Table 3 indicates, not all infrastructural and equipment mandates were given equal weight. Despite guidelines for hot running water onsite, and even apparently for freezers and electric meat saws, inspectors did not take the same hard line on these issues as they took in relation to tiles or glass barriers—especially in rural areas. Another rural/urban difference stemmed from urban by-laws which mandated butchers to use plastic chopping boards. Some urban inspectors insisted these were more “*sanitary and hygienic”* than traditional wooden blocks. It seemed there was either no corresponding requirement in rural areas, or it was not a priority among rural inspectors. Only one rural MI mentioned that some butchers had them and that this represented an improvement.

Infrastructure and equipment at slaughter slabs seemed generally to be of less concern to inspectors. Although four rural MIs and three HOs (two rural and one urban) mentioned telling slab owners to improve infrastructure or checking these structures were kept clean, slab conditions were generally regarded as poor and relatively unchanged, and this enforcement was not described with the same enthusiasm as that applied to butcheries. While some acknowledged the characteristically modest concrete platforms, outfitted with simple drainage systems and sometimes pole-mounted roofs, were indeed improvements over previous practices, a dissatisfaction with the extent of change was also expressed.

*Before cattle were slaughtered on the ground covered with few leaves of bananas and timber, but we advised them to use concrete slabs. However, they are not yet to the recommended standards. Some are dirty, but we ask them to wash the slabs often. So we can say that there are positive changes*. (rural HO)

Four slaughter workers also mentioned that slabs were poor or had not improved. Two of these noted that inspectors were “*more serious with butcheries*” and that they were not pushed to make slab improvements. One worker of 30 years commented:

*The slab structure has not changed at all. The system is still the same. We've been asked to fence the slab but haven't done it yet, we can't afford to. […] Different people from different authorities come here for inspection, they see the situation, and they are satisfied with the way we process meat here. They don't say anything*. (rural slaughter worker)

**Figure 3:**
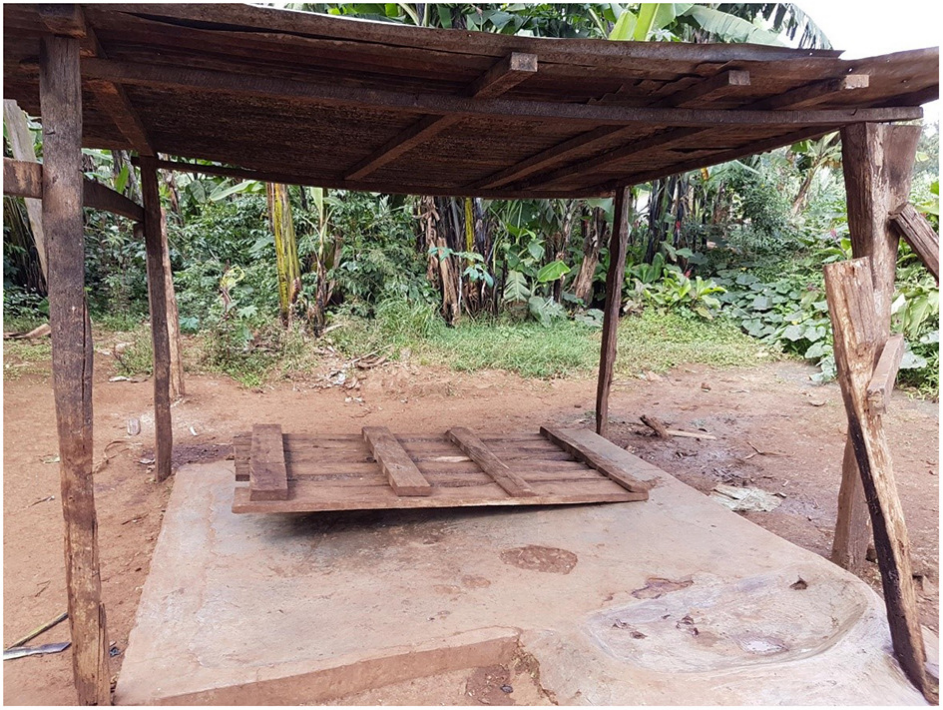
A rural slaughter slab with a pole mounted roof and a wooden pallet. Photo: Linda Waldman.

Despite the comparative lack of perceived change at slabs, six slaughter workers saw their use of wooden pallets—for laying carcasses upon to be skinned or chopped—as improved infrastructure and practice contributing to meat safety (see Figure 3).

*I think the meat is safe now, as we were feeding dirty and unhygienic meat to our customers. Then we said no this is not proper we decided to use better slabs, concrete slabs and wooden pallets*. (rural slaughter worker)

This linking of infrastructure and equipment to meat safety, or at least to general notions of “improvement” in slaughter and meat sale, does suggest understandings and beliefs that link meat safety to aspects such as exposure to “dirty” surfaces, and thus goes beyond the visible presence of abnormalities in animals and meat. And while dirt and dust may be good visible proxies for pathogens, there is no indication in the interviews that respondents were referring to anything other than dirt and dust in their responses. In keeping with the above-described lack of attention to pathogens, most respondents underplayed or overlooked the role of process—how slaughter was performed, meat handled, and infrastructure and equipment kept clean—in mediating meat safety. This is discussed in the next section.

### Meat Handling, Hygiene and Cleanliness

Slaughter workers and MIs tended to emphasize meat inspection and, as discussed above, the former in particular focused on upstream determinants of meat safety (such as livestock keeper practices and conditions at cattle markets). Only one respondent (see Table 2) explicitly linked slaughtering, skinning and chopping to meat safety. However, all slaughter workers reported it was important to keep their work environments “*clean and hygienic”* in a general sense, and six linked this to meat safety.

The details of keeping slaughter slabs and slaughterhouses clean varied from site to site. The public slaughterhouse manager explained that cleaning was conducted continuously throughout the slaughter process, with fresh water being used to constantly wash blood off floors to avoid contamination between carcasses^7^. This respondent did not mention fecal matter or concerns to ensure viscera remained intact during slaughter.

Although none of the slabs had running water onsite (both slaughterhouses did), this was only identified as a challenge by three slaughter workers. Most respondents explained that slabs were brushed down using soap and water carried from a nearby domestic point, only at the beginning and end of a slaughter day. Some slaughter slab workers explained that slabs were simply swept of dirt just prior to slaughter, having been washed after the previous slaughter. All slabs reported usually slaughtering only one animal a day, but many reported processing two or three during periods of higher demand. Although only two slaughter workers described slab cleaning between each animal on these occasions, and only one verbalized the possibility of “*contamination from one animal's meat to another,”* this was not framed as a major concern. Furthermore, one slab worker justified washing the slab between animals, not for hygiene, but for ensuring animal placidity:

*After the first slaughter, we wash the slab before bringing the next animal. This is because if the animal smells the blood of the first, it may become angry and hurt people*. (rural slaughter worker)

The act of cleaning—whatever this entailed—was described as a routine, necessary part of daily business operations, and workers often expressed confidence in their practices. However, only four slaughter slab workers, and both slaughter house workers, mentioned cleaning in response to a question about what they did to ensure meat from their establishment was safe. In this way, cleanliness was less explicitly linked with meat safety than was meat inspection by most slaughter workers.

*I don't have any problems. I perform my duties well. I clean the slab at the end of business. It looks clean as you have seen it today. I am not responsible for ensuring the meat is safe to eat or not. There are people with that obligation, especially the MI*. (rural slaughter worker)

Slaughter workers' confidence in their own cleaning practices was not necessarily shared by all inspectors. HOs, who are responsible for inspecting many types of establishment and often emphasized the importance of cleanliness and hygiene in eateries, claimed to also actively seek assurance that butcheries and slabs were clean and meat handled hygienically. Indeed, they commented on these aspects more frequently than MIs who did not always see enforcement of general hygiene standards to be within their roles, and as discussed above, this is in keeping with their formal remits. This contrast between MIs and HOs is evident in the following quotes.

*[…] after meat inspection, the rest of the work is done by the HO and other staff. I don't want to follow business people that much […]*. (urban MI)

*We also visit butcheries to witness the physical environment and the condition of butchers themselves. How they appear, are they clean, what equipment do they use etc. The meat may have been inspected, but the way it's handled determines its safeness*. (rural HO)

HOs and, to a lesser extent MIs, also expressly associated meat safety with slaughter workers' or butchery staffs' clothing, health and personal hygiene. All HOs claimed to ensure workers had clinically issued health certificates while only one MI mentioned this. “*Very dirty clothes,”* lack of bathing, and human disease were also interpreted as risks to meat safety. Indeed, as evidenced by Table 3, uniforms were of concern to most inspectors. In contrast, while most slaughter workers (*n* = 11) mentioned they were expected to wear uniforms and several reported doing so, only two explicitly linked this to meat safety (Table 4). Rather, they primarily saw this as something inspectors expected of them. Even those that acknowledged dirty clothing as a possible meat safety risk, did not necessarily comply. One described, for instance, the “*dirty clothes and flip flops [sandals]”* that other slaughter workers wore, claiming “*meat contamination starts there.”* Later however, this worker admitted:

*It is required I put on an apron when handling meat, but I ignore this and wear it only when an inspector comes. When they leave, I take it off*. (rural slaughter worker)

Researchers also frequently observed butchers or slaughter workers either not wearing uniforms, or donning them only when prompted by inspectors. During systematic observations, no personnel were seen wearing uniforms.

**Table 4:** Slaughter workers' responses to a specific question asking how they ensured meat safety.

**Ways of ensuring meat safely**	**Slaughter slabs (*n* = 13)**	**Slaughter houses (*n* = 2)**
Reliance on Inspection and the Government Stamp as a guarantee of meat safety	11	2
Ensure the slab/SH is very clean	4	2
Sponge the meat to ensure it is dry/clean	5	0
Wear uniforms	2	0
Avoid contaminants (from the ground, or by covering meat)	1	1
Shorten or delay slaughter date according to an animal's health	1	0
Only slaughter healthy animals	1	0
Observe the organs for signs of disease/abnormality before Inspectors arrive	1	0
Ensure blood is drained away to avoid cross-contamination between animals	0	1

Indeed, and as reflected in Table 2, slaughter workers infrequently suggested that their own slaughtering, skinning and chopping activities might lead to meat contamination when asked what caused meat to become unsafe. Apart from the following exceptional statements from two slaughter workers, one of which was an afterthought at the end of the interview, most did not recognize the role of process and handling as relevant to meat safety.

*Meat can also be contaminated during preparation process such as slaughtering, skinning, chopping, transportation, selling and even food preparation*. (rural slaughter worker)

*Contamination of meat mostly occurs during preparation time, so we have to be careful*. (rural slaughter worker)

Five slaughter workers recognized other pathways to meat contamination. This included dogs, chickens or wild birds accessing the slaughter site although some felt that their cleaning practices were sufficient to mitigate this. At nine of 11 sites, we observed dogs roaming freely. In one slaughterhouse, it was emphasized that carcasses were suspended from railings and “*didn't touch the floor or anything dirty*.” At slabs, some (*n* = 5) explained that skin was removed as animals were suspended. Others (*n* = 8) mentioned that carcasses and meat would be chopped or placed upon an animal's splayed skin, and/or on “*modern wooden pallets”* (described in the previous section and illustrated in Figure 3). These practices were seen as hygienic alternatives to direct contact with the slab surface or ground. One slaughter worker explained how the wooden pallet they used was kept inside his butchery overnight “*to minimize contamination”*; another claimed that while his team did not use one, it was “*a good idea”* which could save skin from damage and “*keep the meat clean*.” Respondents did not, however, talk about cleaning pallets. While this does not necessarily indicate they were not cleaned, it suggests this was not closely associated with meat safety or with daily routine.

Five slaughter workers described “washing,” “drying,” or “clearing” meat of blood (and feces in one case) with a sponge when explaining how they ensured that meat processed at their site was safe to eat.

*We ensure the slaughter slab is very clean. We also wash off blood and faces from the meat using a sponge*. (rural slaughter worker)

*After the animal is skinned and opened, we dry the meat with sponges. The aim is to ensure all blood is removed, and the meat appears clean and attractive. If you wash it using water, the meat will be destroyed and nobody will buy it*. (rural slaughter worker)

**Figure 4:**
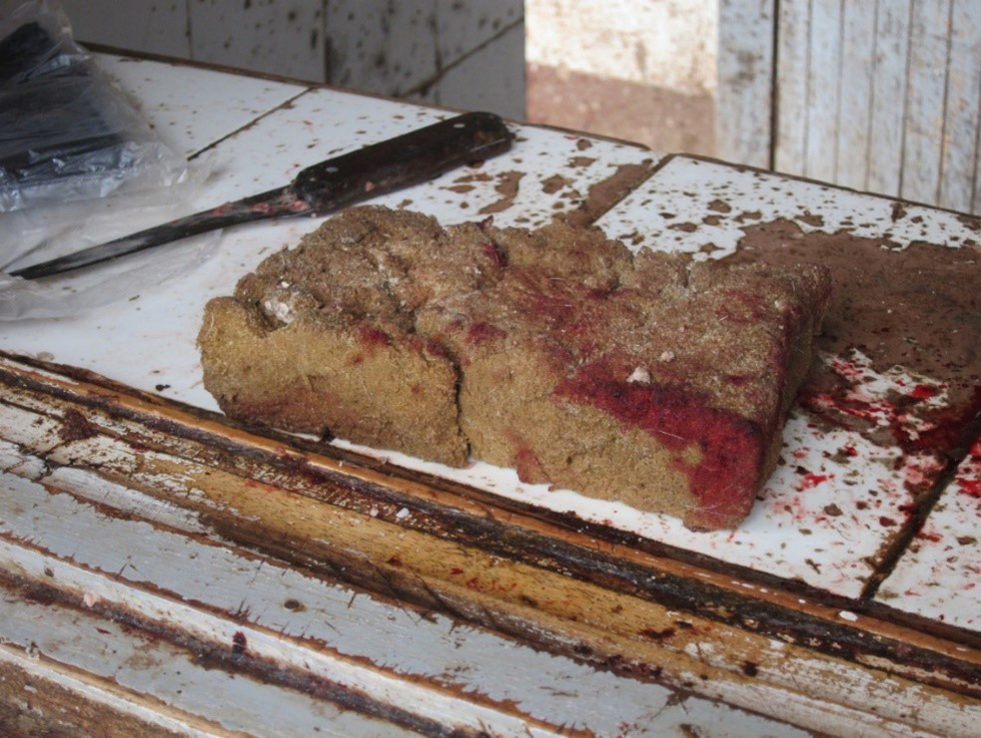
A sponge used to “dry” and “clean” meat on the counter of a rural butchery. Photo: Tabitha Hrynick.

As the second quote suggests, this practice was regarded necessary not only to “clean” meat, but for aesthetic reasons. While one respondent said that sponges were soaked in clean water, no other details were offered regarding whether or how sponges themselves (see Figure 4) were kept clean. Inspectors did not mention the sponges.

Whether, how, and when tools such as knives, uniforms or hands were cleaned seldom figured in the verbalized concerns of all respondents in relation to meat safety, despite regulations mandating this. These practices went widely unmentioned or were downplayed barring a few exceptions. One slaughter worker, for instance, offered the following statement:

*After skinning we have to wash our hands and knives before opening the animal. We cannot use dirty knives to chop meat. There is a lot of sand here, it sticks on the meat, so we have to be careful not to use dirty knives. You cannot remove sand from meat, people won't buy it*. (rural slaughter worker)

In systematic observations at the nine slabs, authors noted workers making efforts to wipe knives with damp cloths or sponges after they had been dropped on the ground surrounding the slab, but not necessarily after being placed on the slab, cutting skin or viscera.

As MIs tended to arrive at slabs for meat inspection after slaughter was complete, they were unable to, and perhaps less interested in, observing the actual process and ensuring, for instance, that tools were cleaned or that care was taken to isolate gut contents and feces. In our systematic observation of the slaughter process, feces were observed on meat at six out of 11 sites (including one slaughterhouse). In two of these instances, workers were observed scraping it off with a knife, and in four, wiping it off with a damp sponge or a cloth.

Handwashing, in contrast to wiping or scraping, was seldom witnessed. Although one slaughter worker noted that the inspector “*reminds us every time he comes here”* to “*keep hands and equipment clean,”* only one MI related handwashing to meat safety in his interview. Although included in the regulations, handwashing was generally not prioritized and, as one urban HO lamented:

…*here we do [animal slaughter] manually with dirty hands on poor slaughter sites. Meat contamination comes from the way the meat is handled, people may have bacterial infections, and consumers will be affected*. (urban HO)

One slaughter worker suggested that handwashing was required, but in general, slaughter workers did not imply it was something they practiced or were inclined to do.

Despite researchers' presence during some interactions between butchery workers and inspectors, and despite the presence of hand washing facilities in most locations (usually suspended buckets with spigots, although not necessarily soap), inspectors did not remark on attendants serving multiple consecutive customers without washing hands or utensils and while handling money, plastic bags, and other objects in addition to meat. As shown above, “cleanliness” and “hygiene” were similarly important to slaughter workers and, although they generally expressed confidence in their practices, they did not always expressly relate them to meat safety. Even when such links were acknowledged, there were indications that breaches to what were considered acceptable standards (whether specified in regulation or shaped by context, social norms and experiential knowledge) were not necessarily seen as problematic enough to take additional biosecurity measures.

## Discussion: Situated Knowledge and Expertise

In this discussion, we argue that inspectors and slaughter workers, with their different incentives, knowledges and priorities, co-construct and enact a situated expertise around meat safety. This situated expertise draws on: technical-scientific understandings of risk as it is rooted in and communicated through regulations and the formal training of inspectors; *and* experiential knowledge and local understandings of disease and risk nested within a particular context of social relations and economic factors related to material and institutional constraints. Indeed, despite conventional understandings of scientific expertise as objective, “all knowledge—including that of science and technology—is situated, partial and embedded” (43). The concept of “situated expertise” encapsulates the manner in which knowledge is contextualized such that it cannot be codified and abstracted from experience and the way in which it is deployed (44).

Despite increasing recognition that scientific expertise should be more attuned to context and local perspectives to enhance its policy relevance (13, 45), little consideration is given to risk managers whose perceptions are shaped both by local understandings and by scientific knowledge. Such work draws on the classic feminist work of Haraway (46) which calls attention to the need to understand what it means for knowledge to be situated, and recommends asking how knowledge operates. This encourages the exploration, not of whether knowledge is objectively true or not, but rather of what the effects of certain kinds of knowledge or pronouncements—believed to be objectively true—might be. In this vein, failing to understand and recognize situated expertise, and the partial knowledges of which it is comprised, resource constraints and the contexts in which people act—and thus relying upon and reifying the superiority of the scientific, the technical and the regulatory—can have the effect of compromising understandings of food safety in both high-income countries and LMICs. Indeed, Cook, drawing on Habermas, argues that “communicative action” and dialogue are necessary to ensure that science can be “untied from the objective claims made” in order to enable the “learning and practice of science” (47). It is through taking account of the multiplicity of partial, situated knowledges that new possibilities for understanding and addressing real-world problems emerge. This challenges the conventional expert-lay binary which conceptually limits *expertise*, in relation to risk, to that which is grounded in technical notions of probability and quantified impact (however measured) to the exclusion of the understandings and flexibility held and practiced by risk managers as they navigate, negotiate and make decisions about risk in real, messy, multi-dimensional contexts. As Corburn (48) argues, conventional “risk-based problem framing and decision-making processes largely ignore evidence that is more informal, experiential, tacit, and explicitly value laden.”

### Area of Consensus: Meat Matters

Despite estimates suggesting *Salmonella* and *Campylobacter* are among the most common causes of FBD in sub-Saharan Africa (5), many respondents demonstrated a preoccupation with anthrax as a singular threat to meat safety. An officially notifiable and widely-known disease in Tanzania (for both humans and animals), anthrax results in sudden animal death and is easily transmitted to humans through contact or consumption of infected animal products. It has serious implications, including death, if treatment is not promptly sought. Parts of northern Tanzania have been designated anthrax “hotspots” due to environmental, social and economic conditions (42, 49). Although it is not always easy to tell if an animal or meat is infected with *Bacillus anthracis*, the cause of anthrax, all inspectors and many slaughter workers were relatively confident in their ability to identify anthrax in animal carcasses and most recalled direct or indirect experiences with it. Respondents' perceptions of the severity of anthrax, and the social reverberations of their and others' experiences with it likely contribute to a “social amplification of risk,” despite in some cases, years-long periods without actually encountering it [c.f. (50)]. This social amplification works to expand the importance of, and to lengthen memories of the disease. Høg et al. used the term “temporalities of risk” to explain risk perceptions in the context of time and experience. Most of their respondents in the Bangladeshi live bird trade did not perceive risk from, or take measures to prevent, avian flu in their stock because they had not heard of or experienced outbreaks in recent years and thus took no biosecurity measures against its prevention (51). In contrast, in Tanzania, despite hearing about occasional isolated anthrax cases, the *absence* of recent widespread or direct experiences, did not temper our respondents' concerns. This may speak to the lasting social and psychological impact of past events, to a high degree of “dread” response to the disease, and to a greater sense of unpredictability and uncontrollability over its emergence (25).

Widespread awareness and concern with anthrax suggests that it has become symbolic of zoonotic and meat-borne disease more broadly for these Tanzanian actors, and it may be contributing to the tendency illustrated in the results to orient attention onto meat itself, and animals' contraction of disease prior to the point of slaughter (and thus, largely outside the control of slaughter workers). Indeed, other conditions of meat with which respondents were primarily concerned were those which were visible, and related to live animals' health conditions. This fosters and reinforces “upstream” understandings of disease and prevailing notions that unsafe meat largely results from poor livestock keeper practices, or conditions and food/water that animals are exposed to during auction at cattle markets and transit.

Leach et al. (52) argue that the ways in which risk is framed have implications for the kinds of mitigation strategies that are seen to be legitimate. Framing meat safety (or lack thereof) as something primarily determined upstream from slaughter, and that can be deduced by sensory observation of animals and meat, legitimates the corresponding—and officialized—strategy of visual inspection and certification by stamp. Unless inspectors equally emphasize the slaughter process itself and how meat is handled thereafter, this upstream and sensory framing serves to obscure the role of slaughter workers in ensuring (or compromising) meat safety, particularly in relation to pathogens and other invisible hazards. Indeed, addressing this is a challenge even in high-income countries where technical risk-based inspection is routinely implemented as the main causes of meat-related FBD continue to be those which are invisible, such as *Campylobacter, Salmonella, Listeria* and *E. coli* O157:H7 (53, 54).

Assessing the appearance of meat is nonetheless a key component and scientifically-justified aspect of meat inspection and safety (including in high-income countries). Furthermore, MIs' focus on hazards—which can be seen, perceived and understood as such by all—over hygiene and process may be a strategic calculation on the part of MIs who see this as a way of maximizing meat safety within their resource-constrained context, and within the parameters of local knowledge, social norms and expectations (36). For their part, slaughter workers were generally deferential to MIs as “*experts*,” respected their judgements, and were open to learning from them. Indeed, many slaughter workers claimed to have learnt from these interactions with MIs how to “*notice if the meat is unsafe to eat by just looking at it.”* This convergence of understanding and priorities represents a shared situated expertise based on a combination of scientific and experiential knowledge that facilitates risk-based decisions about slaughter, sale and condemnation that, to some extent, enhances meat safety.

This emphasis upon animal disease, the appearance of meat and its inspection may indeed play a central role in ensuring that meat purchased in butcheries is free from many pathogens, but it does not address the possible presence of enteric pathogens such as *Salmonella* or *Campylobacter*. Meat can become contaminated with these and other organisms during slaughter and subsequent meat handling, and these can persist on surfaces in slaughter and meat sale environments (55, 56). While, as we show in the next section, the priorities, perceptions and practices of inspectors and slaughter workers do include attention to handling, hygiene and facilitative infrastructure, acceptable standards and perceived risks were variable, unclear, and mediated by the social and economic context (in particular, material and institutional constraints).

### Navigating Risk Management Beyond Meat

Slaughter workers and inspectors shared concerns about visible abnormalities in animals and meat. However, other aspects of meat safety and risk management—such as what counted as adequate or necessary infrastructure, hygiene and associated practice—were less uniformly understood and implemented. Although inspectors had more scientifically-rooted understandings of meat safety—reinforced and embedded in the regulations they were charged with enforcing—they did not necessarily prioritize ensuring that slaughter workers and butchers followed these precisely or all the time. Rather, inspectors were flexible under the circumstances of the real, messy world in which all faced substantial resource constraints, pressures, and differing incentives, and strove to remain sympathetic to the understandings and capacities of slaughter workers in relation to risk management.

MIs' under-emphasis on hygienic practices at slabs and during the slaughter process is partially explained by the fact that they typically arrived after slaughter had actually taken place, due to understaffing and lack of resources for transport. This lack of attention to hygiene might also be explained as a desire to maintain positive relationships with slaughter workers. As suggested by Hrynick et al. (36), trust was a crucial element in these relationships and necessary for keeping slaughter workers “onside” and receptive to inspectors' meat inspection decisions—perceived by all respondents to be of primary importance for meat safety. Additionally, Tanzania's audit into hygiene control in meat production processes in the country revealed a lack of clear, specific criteria for assessing hygiene in meat production (4). This may be a matter of food control authorities having to prioritize activities as they seek to manage a broad array of risks and to align monitoring to their priorities. In our research, this is evident in inspectors' emphasis on meat inspection and compliance with certain infrastructural requirements.

In butcheries, inspectors' focus on ensuring infrastructural compliance in the form of tiles, screens, and windows—aspects clearly in the remit of the butchers/shop owners—reflects their recognition and accommodation of the latter's practical and economic capabilities. In contrast, infrastructural upgrades for which responsibility is more ambiguously shared with the state (running water, electricity) and/or which require substantial investment, went largely unremarked upon as such expectations were considered unreasonable. Similarly, Bardosh et al. (13) noted that Moroccan MIs recognized slaughter workers could not afford to make all regulated infrastructural upgrades and felt morally unable to insist on complete compliance. Furthermore, in focusing on obvious, visible improvements in butcheries, inspectors may be projecting a desire, or perhaps even be responding to political pressure, to publicly demonstrate implementation of regulations in the public interest. In contrast, although several respondents recognized that slaughter slabs were overlooked and would benefit from improved infrastructure, these small, often out-of-sight structures operated outside regular hours, functioning as “backstage” operations, and were relatively invisible to consumers and public scrutiny (57). As such, slaughter workers' perceptions and practices in relation to hygienic processes and meat safety at these sites are of particular significance as they are key actors who have the ability to influence meat safety through their day-to-day activities and practices.

Slaughter workers' understanding of the causes of unsafe meat as “upstream” has the effect of reducing their sense of agency in relation to risk management for meat safety. As illustrated, hygiene practices and infrastructural improvement tend to be regarded as related, but not central, to meat safety (*vis-a-vis* meat inspection). Thus, at times, compliance with some regulations were heeded primarily to satisfy inspectors and not necessarily perceived as means of mitigating meat safety risks. This does not mean that “cleanliness” was not important to slaughter workers, but their notions of what this meant was shaped by how they understood its relevance. Respondents predominantly linked cleaning to notions of appropriate and smooth business operations, respectability and reputational concerns. How clean premises (or meat) “looked” was seen as an indication of quality. This perception may be reinforced and indeed shared with Tanzanian meat consumers, who, Nandonde et al. (38) suggest, have a regulating effect upon the “hygienic beef retailing environment” through their greater patronization of cleaner looking butcheries.

In keeping with this notion of cleanliness as something visible, slaughter workers' concerns were oriented toward observable contaminants like soil on slab surfaces and knives, fecal matter and sand on meat, and keeping meat from coming into contact with the ground (in one slaughterhouse, concern around contamination included animal blood, which was continuously pushed into drains with the aim of mitigating cross-carcass contamination). Thus, slabs were scrubbed and/or brushed prior to and/or after use, tools dropped on the ground were wiped “clean,” and carcasses were suspended where infrastructure would allow. This reveals a widespread understanding about the nature of soil and feces capable of rendering meat unsafe, or at least less sellable. The use of wooden pallets in service of preventing contamination, illustrates a desire to keep meat off the ground, and may suggest a belief in wood, which has been traditionally used for chopping meat, as an appropriate surface on which to dress meat^8^.

Although soil (and fecal matter) are good proxies for the presence of pathogenic bacteria, slaughter workers' notions of cleanliness and contamination seemed to preclude an understanding of pathogens such as *Salmonella* and how they might play a role in spreading them. The example of the sponge, seen as necessary by slaughter workers to “clean” and “dry” meat, demonstrates a disjuncture between pathogen-aware notions of hygiene and those articulated through the situated expertise of slaughter workers, which are reinforced by customers' demand for “clean” looking meat and MIs' lack of attention to meat handling. Given the absence of hot, clean, running water, and no explanations as to how or whether these sponges were themselves kept “clean,” this practice—from a scientific perspective—likely undermines meat safety (59). Nevertheless, this and other practices described above, were in effect, the result of risk-based decision-making, legitimated by a situated expertise involving particular notions of cleanliness, contamination and meat safety, and mediated by available infrastructure and interacting knowledges.

Both MIs' and slaughter workers' relatively relaxed attitudes toward hygiene may also be related to and reinforced by the fact that incidents of FBD are seldom traced back to food, let alone to raw meat, and may instead be attributed to poor cooking or not attributed to food at all. Indeed, one slaughter worker mentioned never having heard of customers becoming ill as a consequence of consuming meat processed at his slab. The challenge of linking FBD to specific foods and origins is not unique to Tanzania, and is also faced in high-income countries (60), although given the complexity and scale of food systems in these contexts, such challenges may be quite different. Nevertheless, health systems and food surveillance systems in Tanzania and other LMICs are weak and people may not attribute their own experiences of FBD (such as diarrhea) to food, nor seek medical attention (7, 61, 62). Furthermore, eatery operators have reported cooking meat for long periods which raises questions about whether meat is actually a significant source of FBD in Tanzania and thus about the appropriate degree of regulation and intervention. Similarly, although there were concerns about some Tanzanian populations consuming raw meat (49, 63), inspectors, and HOs in particular, mentioned their efforts to remind people to cook meat for long periods. Local practices of doing just this reflect another form of situated expertise held by communities, informed by experience and practical wisdom adapted to life with limited electricity, and capacity to preserve food.

HOs also, for their part, emphasized meat handling and hygiene (of infrastructure, equipment and personnel) and its link with meat safety much more than MIs. However, slaughter workers made far fewer references to these issues and it was unclear how frequently HOs visited slaughter sites as they have many other duties, and unlike MIs, do not necessarily make such visits as a matter of routine. In contrast, and as described above, slaughter workers were clearly highly influenced by MIs who were more inclined to overlook questions of meat handling and hygiene, sometimes seeing this as beyond their remits. Despite indications that MIs and HOs often support each other and at times play overlapping roles given resource constraints, there is no clear mechanisms for encouraging such collaboration and support. There may thus be a case for introducing policy which closes the apparent gap between their remits (both official and perceived) and practices, and strengthens the assemblage of partial knowledges upon which decisions about meat safety and associated risk are made.

## Conclusions

Through their interactions, inspectors and slaughter workers co-construct and enact a situated expertise to manage risk in relation to meat safety. While informed by scientific, technical risk knowledge, this expertise is also shaped by local logics and contextual conditions which bring into consideration material and institutional constraints, local expectations of appropriate business operation and consumer demand, and perceptions of hygiene, cleanliness and meat quality. This interaction between scientific and experiential knowledge can enhance meat safety. For instance, slaughter workers and inspectors uniformly agree that meat should be inspected and condemned if it exhibits visibly problematic signs, while slaughter workers' concern with soil, feces and maintaining a “clean” environment can help to reduce the risks associated with pathogen contamination.

At the same time, the diverse priorities and practices of different respondents may compromise meat safety. Workers' experiential knowledge and inspectors' (especially MIs') under-emphasis of process and hygiene is at odds with meat safety (and the technical risk paradigm), particularly when hazards affecting meat are not immediately obvious or amenable to redress with available resources. The concern with what is visible, and the preclusion of contaminating pathogens legitimates potentially compromising practices such as wiping soil and feces off meat and tools and the prioritization of public, visible sites of meat production (butcheries) over more private and inconspicuous ones (slaughter slabs). This is reinforced by a lack of feedback connecting ill-health to the process of meat production at the local level. Conversely, HOs' greater concern with hygiene issues, but seemingly limited contact with slaughter workers and sites (alongside MIs' reservations about addressing hygiene), may represent an opportunity to strengthen meat safety at the local level through a more multi-sectoral approach which more explicitly emphasizes context-appropriate hygiene practices and improvements.

It is clear, from the above results and discussion that, in the absence of financial, technological, and scientific investment to revolutionize meat production in LMICs, both technical risk-based knowledge, and situated expertise must be taken into account. Local understandings and practices must be taken seriously as opposed to being seen as evidence of a knowledge deficit, a failure to assess and respond to risk, or as forms of cultural conservatism (13, 64). Exclusive reliance upon technical knowledge, either in an attempt to regulate meat safety or to assess meat safety implementation, will continue to run up against inspectors' and slaughter workers' situated expertise as practice and practicality take priority over theory. Consequentially, this can compromise meat safety or make inspection and regulation, as it plays out in real life, appear profoundly deficient.

Taking situated expertise for risk management seriously involves, as this paper has shown, acknowledging the logics and understandings behind inspectors' and slaughter workers' priorities and practices for meat safety alongside recognition of the particular social, economic and institutional conditions in which they work. It further requires facilitating shared expertise between these actors, in relation to the ways contaminating pathogens are transmitted and spread, and highlighting the importance of hence under-emphasized processes and places associated with meat production. Such encouragement, facilitation and adoption of context-appropriate, multi-sectoral solutions will enable slaughter workers and inspectors to more clearly recognize their own agency in relation to meat safety, collaborate more closely, address the gaps between their roles and responsibilities and take more informed risk-based decisions, predicated on a situated expertise that embraces a broader assemblage of partial knowledges.

The research has the following limitations: qualitative research is time consuming, can be costly and does not seek to ensure representative samples. For these reasons, sample sizes tend to be small and this study is not representative of all slaughter workers in Tanzania. This research is based on qualitative interviews, which included a combination of structured open-ended questions and, in follow-up to each of these questions, relatively unstructured discussion and, interaction. Data saturation was achieved in terms of interviews no longer yielding substantive new information nor new codes; detailed and nuanced data descriptions having been attained (65) and the data having provided substantial insights which theoretically informed the research questions (66). Nonetheless, as the researchers involved in the interviewing assumed a “role of research instrument” (67) through follow-up questioning and discussion, it may be challenging to replicate these findings. As mentioned above, this research forms part of HAZEL, a larger, multi-disciplinary project to understand hazards associated with zoonotic enteric pathogens in emerging livestock meat pathways. Future research will seek to integrate this qualitative assessment of risk perception into a Bayesian belief network (BBN), a form of probabilistic graphical model that allows for integration of information from disparate sources. This will provide information regarding the risks associated with foodborne pathogens in meat supply chains in Tanzania, and show how decisions to optimize food safety are influenced by information supply when economic factors cause changes in patterns of beef production and consumption. Future research should also investigate the molecular epidemiology of foodborne illness in Tanzania and other LMICs.

## Data Availability Statement

The datasets used and/or analyzed during the current study are currently available from the corresponding author on reasonable request, and will be lodged in a public repository at the culmination of the project.

## Ethics Statement

The studies involving human participants were reviewed and approved by The Tanzanian National Institute of Medical Research (Ref. NIMR/HQ/R.8a/Vol. IX/2028 and extension Ref. NIMR/HQIR.8cNol. 11/1069); the Kilimanjaro Christian Medical Centre (KCMC) Ethics Committee (Research Ethical Certificate No. 832); the Ethics Committee of the College of Medical, Veterinary and Life Sciences at the University of Glasgow, Glasgow, UK (Refs. 200140183 and 200140152) and the Human Research Ethics Committee at the University of Otago, Dunedin, New Zealand (Ref. H15/069). Written informed consent was not provided because, in keeping with the Framework for Research Ethics produced by the UK Economic and Social Research Council, our ethical applications stressed that, in order to ensure no-one is made to feel inadequate where participants are not literate, or in situations where researchers are unsure of the literacy status of respondents, verbal consent would be obtained.

## Author Contributions

BMm, ES, GP, JC, JS, KT, LW, NM-M, and RZ contributed to the designing of the research and field study. BMa conducted all interviews, translation, and transcription. LW and TH were responsible for literature review, data analysis, formulating key arguments, and drafting the manuscript. BMa, ES, GP, JC, JB, JS, KT, MD, RZ, and SC contributed to the manuscript's critical revision and intellectual development.

## Conflict of Interest

The authors declare that the research was conducted in the absence of any commercial or financial relationships that could be construed as a potential conflict of interest.

## Footnotes

^1^Slaughterhouses, characterized as roofed or enclosed buildings, are larger than slaughter slabs with more systematic and formal operating procedures, infrastructure (drainage systems and running water) and equipment (such as hooks). Abattoirs are urban and are the most formal and mechanized facilities in Tanzania (4). The Tanzania Food and Drugs Authority records that there are 1,084 slaughter slabs and 85 slaughterhouses in Tanzania (4). In Moshi municipality and Moshi district, there are five slaughterhouses and an estimated 260 slaughter slabs.

^2^Due to resource constraints, ongoing training is not typical and thus continuing formal communication to inspectors about what constitutes “risk” and what should be done to manage it happens primarily through legislation and policy communicated in meetings and via communiques. MIs generally have a certificate or diploma in animal health, and may have taken optional courses on HACCP-style risk-based approaches (personal comms. Sindiyo). Similarly, HOs hold certificates or diplomas in environmental health, and some in our study reported having studied meat safety specifically. The Ministry of Health and Social Work estimated, in 2012, that there were 2047 Environmental Health Officers and Assistant Officers in Tanzania. Of these, 94 worked in the Kilimanjaro region, within which Moshi falls (31). The Department of Veterinary Services reported, in 2014, that there were 1,350 MIs in Tanzania, although only about half of these were fully qualified. These are “assigned to the slaughter slabs of Regions and Districts” (32). In Moshi municipality and Moshi district, there are an estimated 40 HOs and 56 MIs.

^3^Both HOs and MIs are employed directly by Local Government Authorities, but have technical remits under different government authorities. While HOs answered to the Ministry of Health and the Tanzania Food and Drug Authority (TFDA) during the time of the research, MIs are accountable to the Ministry of Livestock and Fisheries Development. Technically, MIs are responsible for ante-mortem and post-mortem inspection before and immediately after slaughter. Thereafter, the product is considered to be food and its safety (including handling, transportation, and storage) becomes the responsibility of the HO.

^4^Signs commonly associated with anthrax in dead animals include dark blood that does not clot, bleeding from the mouth, nose, and other orifices, subcutaneous swelling, rapid bloating and an absence of rigor mortis. In live animals, “fever, dyspnea, agitation, and convulsions” occur before sudden death (42).

^5^The Tanzania Food and Drug Authority (TFDA) Act of 2003, The Animal Disease Act of 2003, the Meat Industry Act of 2006 and the Public Health Act of 2009.

^6^Respondents used the term “directives” in a general sense, implying that these came “from above.” Respondents may have been generally referencing all relevant legislation at all levels, or more specific local bylaws, national regulations, or *ad-hoc* orders from ministries or other authorities.

^7^Unlike at slabs, multiple animals were slaughtered each day at the slaughterhouses.

^8^This practice at several rural slabs was happening concurrently alongside efforts in urban wards to ban the use of wooden chopping boards in butcheries for reasons of hygiene. There is a lack of scientific consensus on the superiority of plastic chopping surfaces *vis a vis* wooden ones (58).

## References

[1] Wilson RT The Red Meat Value Chain in Tanzania: A Report from the Southern Highlands Food Systems Programme. FAO (2015). Available online at: http://www.fao.org/sustainable-food-value-chains/library/details/en/c/285410/ (accessed June 10, 2019).

[2] Michael S Mbwambo N Mruttu H Dotto M Ndomba C da Silva M Tanzania Livestock Master Plan. Nairobi: International Livestock Research Institute (ILRI) (2018).

[3] MLFD. Tanzania Livestock Modernization Initiative. Dar es Salaam: Ministry of Livestock and Fisheries Development (2015).

[4] URT A Performance Audit Report on the Hygiene Control in Meat Production. Dar es Salaam: United Republic of Tanzania (2016).

[5] Havelaar AH Kirk MD Torgerson PR Gibb HJ Hald T Lake RJ. World Health Organization global estimates and regional comparisons of the burden of foodborne disease in 2010. PLoS Med. (2015) 12:e1001923. 10.1371/journal.pmed.1001923 26633896 PMC4668832

[6] Jaffee S Henson S Unnevehr L Grace D Cassou E The Safe Food Imperative: Accelerating Progress in Low-and Middle-Income Countries. The World Bank (2018). Available online at: https://openknowledge.worldbank.org/bitstream/handle/10986/30568/9781464813450.pdf?sequence=6 (accessed March 19, 2019).

[7] Roesel K Grace D Food Safety and Informal Markets: Animal Products in Sub-Saharan Africa. Oxon: Routledge (2015).

[8] Zanin LM da Cunha DT de Rosso VV Capriles VD Stedefeldt E. Knowledge, attitudes and practices of food handlers in food safety: an integrative review. Food Res Int. (2017) 100:53–62. 10.1016/j.foodres.2017.07.042 28873718

[9] Douglas M Wildavsky AB Risk and Culture: An Essay on the Selection of Technical and Environmental Dangers. Berkeley, CA: University of California Press (1982).

[10] Palinkas LA Horwitz SM Green CA Wisdom JP Duan N Hoagwood K. Purposeful sampling for qualitative data collection and analysis in mixed method implementation research. Adm Policy Ment Health. (2015) 42:533–44. 10.1007/s10488-013-0528-y 24193818 PMC4012002

[11] Havelaar AH Brul S De Jong A De Jonge R Zwietering MH Ter Kuile BH. Future challenges to microbial food safety. Int J Food Microbiol. (2010) 139:S79–94. 10.1016/j.ijfoodmicro.2009.10.015 19913933

[12] Sjölander-Lindqvist A Cinque S When wolves harm private property. Focaal. (2013) 65:114–28. 10.3167/fcl.2013.650110

[13] Bardosh KL El Berbri I Ducrotoy M Bouslikhane M Ouafaa F Welburn SC. Zoonotic encounters at the slaughterhouse: pathways and possibilities for the control of cystic echinococcosis in northern Morocco. J Biosoc Sci. (2016) 48:S92–115. 10.1017/S0021932015000486 27428068

[14] Beck U Risk Society: Towards a New Modernity. London: Sage (1992).

[15] Sears A Baker MG Wilson N Marshall J Muellner P Campbell DM. Marked campylobacteriosis decline after interventions aimed at poultry, New Zealand. Emerg Infect Dis. (2011) 17:1007. 10.3201/eid/1706.101272 21749761 PMC3358198

[16] EFSA Meat Inspection: MAKING RISK a Factor in Meat Inspections. (2012). Available online at: https://www.efsa.europa.eu/en/press/news/120711f (accessed December 3, 2018).

[17] O'Brien SJ. The “decline and fall” of nontyphoidal Salmonella in the United Kingdom. Clin Infect Dis. (2012) 56:705–10. 10.1093/cid/cis967 23166188 PMC3563394

[18] Taylor E Taylor J Perceptions of “the bureaucratic nightmare” of HACCP: A case study. Br Food J. (2004) 106:65–72. 10.1108/00070700410515217

[19] Grace D. Food Safety in Developing Countries: Research Gaps and Opportunities. White Paper (2017). Available online at: https://cgspace.cgiar.org/bitstream/handle/10568/81515/White%2520paper%2520food%2520safety.pdf?sequence=1 (accessed June 10, 2019).

[20] Vipham JL Chaves BD Trinetta V. Mind the gaps: how can food safety gaps be addressed in developing nations? Anim Front. (2018) 8:16–25. 10.1093/af/vfy020 32002226 PMC6951884

[21] WHO Food Safety and Nutrition Food Law Guidelines (n.d.). Available online at: https://afro.who.int/publications/food-safety-and-nutrition-food-law-guidelines (accessed December 4, 2018).

[22] FAO and WHO Food Safety Risk Analysis: A Guide for National Food Safety Authorities. Food and Nutrition Paper 87. Food and Agriculture Organization and World Health Organization (2006). Available online at: http://www.fao.org/docrep/012/a0822e/a0822e00.pdf (accessed December 4, 2018). 17891885

[23] Renn O Jaeger CC Rosa EA Webler T The rational actor paradigm in risk theories: Analysis and critique. In: Cohen E, editor. Risk in the Modern Age. London: Palgrave Macmillan 2000 p. 35–61.

[24] Tulloch J Lupton D Risk and Everyday Life. London: Sage (2003).

[25] Jensen KK Lassen J Robinson P Sandøe P. Lay and expert perceptions of zoonotic risks: understanding conflicting perspectives in the light of moral theory. Int J Food Microbiol. (2005) 99:245–55. 10.1016/j.ijfoodmicro.2004.09.004 15808359

[26] McDonald MA Kucera KL. Understanding non-industrialized workers' approaches to safety: How do commercial fishermen “stay safe”? J Safety Res. (2007) 38:289–97. 10.1016/j.jsr.2006.10.009 17617238

[27] Waldman L The Politics of Asbestos: Understandings of Risk, Disease and Protest. London: Routledge (2011).

[28] Renn O Rohrmann B (eds). Cross-Cultural Risk Perception: A Survey of Empirical Studies. Dordrecht: Springer Science and Business Media (2000).

[29] Waldman L Urbanisation, the Peri-Urban Growth and Zoonotic Disease. (2015). Available online at: http://opendocs.ids.ac.uk/opendocs/handle/123456789/5855 (accessed June 10, 2019).

[30] Long N Development Sociology: Actor Perspectives. London: Routledge (2001).

[31] MHSW Human Resource for Health Country Profile 2012/13. Dar es Salaam: Ministry of Health and Social Welfare (2013).

[32] Stemshorn B Carron M Munstermann S Wakhusama S PVS Evaluation Follow-UP Mission Report: Tanzania. Report of the Veterinary Services of Tanzania. OIE, France (2016).

[33] Lipsky M Street-Level Bureaucracy: Dilemmas of the Individual in Public Services. New York, NY: Sage Publications (1980).

[34] Gilson L Lipsky's Street Level Bureaucracy. In: Lodge M Balla S, editors. Oxford Handbook of the Classics of Public Policy. Oxford: Oxford (2015). p. E.

[35] Funder M Marani M Local bureaucrats as bricoleurs. The everyday implementation practices of county environment officers in rural Kenya. Int J Commons. (2015) 16:87–106. 10.18352/ijc.526

[36] Hrynick TA Barasa V Benschop J Cleaveland S Crump JA Davis M. Street-level diplomacy and local enforcement for meat safety in northern Tanzania: knowledge, pragmatism and trust. BMC Public Health. (2019) 19:863. 10.1186/s12889-019-7067-8 31269927 PMC6610827

[37] Boholm Å Henning A Krzyworzeka A Anthropology and decision making. Focaal. (2013) 65:97–113. 10.3167/fcl.2013.650109

[38] Nandonde S Elibariki M Mtenga L Assessment on economic support and value of hygiene of butcher shops among beef consumers in Tanzania. Assessment. (2012) 3:13.

[39] Baker D Mtimet N Pica-Ciamara U Nsiima L Consumer's preferences for animal source foods and retail outlets: the Case of Tanzania. Afr J Agric Resourc Econ. (2016) 11:197–210. 10.22004/ag.econ.245939

[40] Hartmann C Hübner P Siegrist M. A risk perception gap? Comparing expert, producer and consumer prioritization of food hazard controls. Food Chem Toxicol. (2018) 116:100–7. 10.1016/j.fct.2018.04.006 29626580

[41] Saldaña J The Coding Manual for Qualitative Researchers. Sage: London (2016).

[42] Mwakapeje ER Justine AA Kunda JS Mjingo EE Makondo ZE Nonga HE Prevention, detection, and response to anthrax outbreak in Northern Tanzania using one health approach: a case study of Selela Ward in Monduli District. Int J One Health. (2017) 3:66–76. 10.14202/IJOH.2017.66-76

[43] Leach M Waldman L Centres of Excellence? Questions of Capacity for Innovation, Sustainability, Development. (2012). Available online at: https://opendocs.ids.ac.uk/opendocs/ds2/stream/?#/documents/8112/page/1 (accessed June 10, 2019).

[44] Luntley M Knowing how to manage: expertise and embedded knowledge. Reason Pract. (2002) 2:3–14. 10.5840/pom20022311

[45] Campbell N Reconstructing science and technology studies: views from feminist standpoint theory. Front J Women Stud. (2009) 30:1–29. 10.1353/fro.0.0033

[46] Haraway D Situated knowledges: the science question in feminism and the privilege of partial perspective. Feminist Stud. (1988) 14:575–99. 10.2307/3178066

[47] Cook K Grappling with wicked problems: exploring photovoice as a decolonizing methodology in science education. Cult Stud Sci Educ. (2015) 10:581–92. 10.1007/s11422-014-9613-0

[48] Corburn J Street Science: Community Knowledge and Environmental Health Justice. Cambridge, MA: MIT Press (2005).

[49] Mangesho PE Neselle MO Karimuribo ED Mlangwa JE Queenan K Mboera LEG. Exploring local knowledge and perceptions on zoonoses among pastoralists in northern and eastern Tanzania. PLoS Negl Trop Dis. (2017) 11:e0005345. 10.1371/journal.pntd.0005345 28146556 PMC5325590

[50] Kasperson RE Renn O Slovic P Brown HS Emel J Goble R The social amplification of risk: a conceptual framework. Risk Anal. (1988) 8:177–87. 10.1111/j.1539-6924.1988.tb01168.x

[51] Høg E Fournié G Hoque MA Mahmud R Pfeiffer DU Barnett T Competing biosecurity and risk rationalities in the Chittagong poultry commodity chain, Bangladesh. BioSocieties. (2019) 14:368–392. 10.1057/s41292-018-0131-2

[52] Leach M Stirling AC Scoones I Dynamic Sustainabilities: Technology, Environment, Social Justice. Routledge: London (2010).

[53] Wagenaar JA French NP Havelaar AH. Preventing Campylobacter at the source: why is it so difficult? Clin Infect Dis. (2013) 57:1600–6. 10.1093/cid/cit555 24014733

[54] Chlebicz A Slizewska K. Campylobacteriosis, salmonellosis, yersiniosis, and listeriosis as zoonotic foodborne diseases: a review. Int J Environ Res Public Health. (2018) 15:E863. 10.3390/ijerph15050863 29701663 PMC5981902

[55] De Cesare A Sheldon BW Smith KS Jaykus LA. Survival and persistence of Campylobacter and Salmonella species under various organic loads on food contact surfaces. J Food Prot. (2003) 66:1587–94. 10.4315/0362-028X-66.9.1587 14503710

[56] Leotta GA Brusa V Galli L Adriani C Linares L Etcheverría A. Comprehensive evaluation and implementation of improvement actions in butcher shops. PLoS ONE. (2016) 11:e0162635. 10.1371/journal.pone.0162635 27618439 PMC5019392

[57] Rheinländer T Olsen M Bakang JA. Keeping up appearances: perceptions of street food safety in Urban Kumasi, Ghana. J Urban Health. (2008) 85:952. 10.1007/s11524-008-9318-3 18821020 PMC2587647

[58] Cliver DO. Cutting boards in Salmonella cross-contamination. J AOAC Int. (2006) 89:538–42. 10.1093/jaoac/89.2.538 16640304

[59] Wolde T Bacha K. Microbiological safety of kitchen sponges used in food establishments. Int J Food Sci. (2016) 2016:1–7. 10.1155/2016/1659784 27840819 PMC5093261

[60] Hoffmann S Devleesschauwer B Aspinall W Cooke R Corrigan T Havelaar A Attribution of global foodborne disease to specific foods: Findings from a World Health Organization structured expert elicitation. PLoS One. (2017) 12 10.1371/journal.pone.0183641 PMC5598938 28910293

[61] Nonga HE Sells P Karimuribo ED. Occurrences of thermophilic campylobacter in cattle slaughtered at morogoro municipal Abattoir, Tanzania. Trop Anim Health Prod. (2010) 42:73–8. 10.1007/s11250-009-9387-7 19551483

[62] Grace D. Food safety in low and middle income countries. Int J Environ Res Public Health. (2015) 12:10490–507. 10.3390/ijerph120910490 26343693 PMC4586623

[63] Swai ES Schoonman L Daborn C. Knowledge and attitude towards zoonoses among animal health workers and livestock keepers in Arusha and Tanga, Tanzania. Tanz J Health Res. (2010) 12:272–7. 10.4314/thrb.v12i4.54709 24409636

[64] Hansen J Holm L Frewer L Robinson P Sandøe P. Beyond the knowledge deficit: recent research into lay and expert attitudes to food risks. Appetite. (2003) 41:111–21. 10.1016/S0195-6663(03)00079-5 14550309

[65] Fusch PI Ness LR Are we there yet? Data saturation in qualitative research. Qual Rep. (2015) 20:1408–16. Available online at: https://cpb-us-e1.wpmucdn.com/sites.nova.edu/dist/a/4/files/2015/09/fusch1.pdf

[66] Sanders B Sim J Kingston T Baker S Waterfield J Bartlam B. Saturation in qualitative research: Exploring its conceptualization and operationalization. Quant Qual. (2018) 52:1893–907. 10.1007/s11135-017-0574-8 29937585 PMC5993836

[67] Aguinis H Solarino AM Transparency and replicability in qualitative research: the case of interviews with elite informants. Strat Mgmt J. (2019 40:1921–315. 10.1002/smj.3015

